# Aqueous humor tyrosinase activity is indicative of iris melanocyte toxicity

**DOI:** 10.1016/j.exer.2017.07.006

**Published:** 2017-09

**Authors:** Sarmistha Mahanty, Ankush A. Kawali, Shruthi Shirur Dakappa, Padmamalini Mahendradas, Mathew Kurian, Varun Kharbanda, Rohit Shetty, Subba Rao Gangi Setty

**Affiliations:** a Department of Microbiology and Cell Biology, Indian Institute of Science, Bangalore 560012, India; b Department of Uveitis and Ocular Immunology, Narayana Nethralaya, Bangalore 560010, India; c Department of Cataract, Narayana Nethralaya, Bangalore 560010, India; d Department of Cornea and Refractive Surgery, Narayana Nethralaya, Bangalore 560010, India

**Keywords:** Melanin, Dispersed pigments, Tyrosinase, Soluble tyrosinase, L-DOPA, Fluoroquinolone, Moxifloxacin, Ciprofloxacin, Tobramycin, BAIT and BADI, FQL, fluoroquinolone, TYR, tyrosinase, TYR^M^, mushroom tyrosinase, L-DOPA, L-3,4-dihydroxyphenylalanine, AC tap, anterior chamber tap, BAK, benzalkonium chloride, BADI, bilateral acute depigmentation of the iris, BAIT, bilateral acute iris transillumination

## Abstract

Antibiotics such as fluoroquinolones (FQLs) are commonly used to treat ocular infections but are also known to cause dermal melanocyte toxicity. The release of dispersed pigments from the iris into the aqueous humor has been considered a possible ocular side effect of the systemic administration of FQLs such as Moxifloxacin, and this condition is known as bilateral acute iris transillumination (BAIT). Bilateral acute depigmentation of iris (BADI) is a similar condition, with iris pigment released into the aqueous, but it has not been reported as a side effect of FQL. Iris pigments are synthesized by the melanogenic enzyme tyrosinase (TYR) and can be detected but not quantified by using slit-lamp biomicroscopy. The correlation between dispersed pigments in the aqueous and the extent of melanocyte toxicity due to topical antibiotics *in vivo* is not well studied. Here, we aimed to study the effect of topical FQLs on iris tissue, the pigment release in the aqueous humor and the development of clinically evident iris atrophic changes. We evaluated this process by measuring the activity of TYR in the aqueous humor of 82 healthy eyes undergoing cataract surgery following topical application of FQLs such as Moxifloxacin (27 eyes, preservative-free) or Ciprofloxacin (29 eyes, with preservative) or the application of non-FQL Tobramycin (26 eyes, with preservative) as a control. In addition, the patients were questioned and examined for ocular side effects in pre- and post-operative periods. Our data showed a significantly higher mean TYR activity in the aqueous humor of Ciprofloxacin-treated eyes compared to Moxifloxacin- (preservative free, *p* < 0.0001) or Tobramycin-treated eyes (*p* < 0.0001), which indicated that few quinolones under certain conditions are toxic to the iris melanocytes. However, the reduced TYR activity in the aqueous of Moxifloxacin-treated eyes was possibly due to the presence of a higher drug concentration, which inhibits TYR activity. Consistently, immunoblotting analysis of the aqueous humor from both Ciprofloxacin- and Moxifloxacin-treated eyes showed the presence of soluble TYR enzyme, thus reflecting its toxicity to iris melanocytes and corresponding to its activity in the aqueous humor. Intriguingly, none of these patients developed any clinically appreciable ocular side effects characteristic of BAIT or BADI. Overall, our results suggest that topical antibiotics cause different levels of iris melanocyte toxicity, releasing dispersed pigments into the aqueous humor, which can be measured through TYR enzyme activity. Hence, we conclude that topical FQLs may cause subclinical toxicity to the iris melanocytes but may not be the sole cause of the development of BAIT or BADI.

## Highlights

• Exposure to antibiotics results in release of dispersed pigments into the aqueous humor due to iris melanocyte toxicity.
• Melanins or dispersed pigments in the aqueous humor are quantifiable by using an L-DOPA based TYR activity assay.
• Soluble TYR contributes to the activity in aqueous and is inhibited by higher drug concentrations.
• Treatment with a fluoroquinolone alone may not cause any acquired iris atrophy diseases.

## 1 Introduction

Melanin biopolymers are synthesized by melanocytes in the unique organelles called melanosomes (Raposo and Marks, 2007, Sitaram and Marks, 2012). Melanocytes in skin, hair and eyes provide color and photoprotection. An inhibition of melanin synthesis in melanocytes by genetic alteration or mutation results in oculocutaneous albinism (Jani et al., 2016, Marks et al., 2013). However, exposure to medications such as fluoroquinolone (FQL) antibiotics causes phototoxicity in skin melanocytes, possibly by inhibiting melanization (Beberok et al., 2011, Beberok et al., 2015a, Beberok et al., 2015b), but this mechanism is not well studied in iris melanocytes. Moreover, melanin deposits and melanocyte debris, which are collectively called dispersed pigments, are commonly observed in the aqueous humor of the eye during iris atrophic conditions such as bilateral acute iris transillumination (BAIT) and bilateral acute depigmentation of the iris (BADI) (Goktas and Goktas, 2011, Tugal-Tutkun et al., 2009, Tugal-Tutkun and Urgancioglu, 2006). It is not clear how these dispersed pigments accumulate in the aqueous humor (anterior chamber) of the eye since the mechanism of pathogenesis of these diseases is still unknown. Interestingly, a few studies implied that the systemic use of FQL antibiotics such as Moxifloxacin causes BAIT (Bringas Calvo and Iglesias Cortinas, 2004, Hinkle et al., 2012, Wefers Bettink-Remeijer et al., 2009, Willermain et al., 2010), wherein red eyes, photophobia, dispersed pigments in the aqueous and iris atrophic changes are observed (Tugal-Tutkun et al., 2011). In contrast, studies have also suggested a viral etiology for these conditions (Tugal-Tutkun et al., 2009). Nevertheless, the relation between topical Moxifloxacin treatment and the pathogenesis of BAIT or BADI has never been tested, and the possibility cannot be excluded. We hypothesized that the accumulation of dispersed pigments in these diseases is due to the ocular side effects or phototoxicity of iris melanocytes from FQL antibiotics. This process notably increases the release of dispersed pigments, which include melanin synthesizing enzyme, tyrosinase (TYR). In general, slit-lamp biomicroscopy is used to detect these pigment deposits in the anterior chamber of the eye (Tugal-Tutkun et al., 2011), but it is not a quantitative method of measuring the extent of melanocyte damage. Here, we have developed a method for measuring melanocyte toxicity *in vivo* by estimating the TYR activity in the aqueous humor. Additionally, we have studied the direct effect of FQLs on iris melanocytes. Our studies show that both Ciprofloxacin (with preservative) and Moxifloxacin (preservative free) treatments are toxic to the iris melanocytes, as indicated by the presence of soluble TYR enzyme and a dramatic increase in TYR activity in the aqueous of Ciprofloxacin- but not Moxifloxacin-treated eyes. Furthermore, the reduced TYR activity in the aqueous of Moxifloxacin-treated eyes is possibly due to the presence of higher concentration of Moxifloxacin, which inhibits aqueous TYR activity. Overall, our studies provide the first evidence that the topical application of FQL causes subclinical iris melanocyte toxicity but that conditions such as BAIT/BADI may not develop without other contributory factors.

## 2 Materials and methods

To investigate the effect of topical FQLs on iris melanocytes and the pathogenesis of BAIT/BADI, we chose healthy eyes of patients undergoing routine cataract surgery (Phacoemulsification). This is a prospective, interventional case study of 82 eyes of 82 patients undergoing cataract surgery from August 2015 to May 2016. The study was approved by an internal review board and adhered to the declarations of Helsinki. All cataract patients in our study did not have any ocular disease/defects prior to the surgery. We divided the patients (both male and female) into 3 groups. Group A (*n* = 27) received the FQL antibiotic Moxifloxacin (Vigamox^®^, Alcon Laboratories, Fort Worth, TX, USA; preservative-free), Group B (*n* = 29) received another FQL antibiotic Ciprofloxacin (Ciplox^®^, Cipla Ltd., Ahmedabad, India; with preservative) and Group C (*n* = 26) received the non-FQL antibiotic Tobramycin (Tobrex^®^, Alcon Laboratories, manufactured by Wintac Ltd., Bangalore, India; with preservative) as a control to FQL antibiotic. The demographic information of these patients includes the following: a female average age of 39 Y (Gr. A: 12, Gr. B: 15, Gr. C: 12) and a male age range of 34–87 Y (Gr. A: 62.52, Gr. B: 63.64, Gr. C: 63.57). All groups were treated with the respective antibiotic topically 4 times/day for 3 days as preoperative medication before planned cataract surgery. All patients underwent routine eye examination. An AC (anterior chamber) tap was done on the table just before starting the surgery. A volume of 0.1–0.25 cc (cubic centimetre) aqueous was collected for the measurement and analysis of dispersed pigments. Samples were examined using a slit-lamp for the presence of dispersed pigments and were then stored at 4 °C (^0^C) before estimating the aqueous TYR activity (see below), which was done within 3 days for different batches of samples. Thereafter, we followed up the patients in the post-operative period and looked for signs of BAIT/BADI.

In this study, we included healthy eyes with cataract only and excluded (1) cases with pigment dispersion syndrome or pigment dispersion post dilatation, patients with a history of ocular trauma and cases with iris or choroidal nevus; (2) patients who had been treated with oral or topical antibiotics (except preoperative medications) in the past month; and (3) patients on medications that interfered with dopamine because L-DOPA (L-3,4-dihydroxyphenylalanine) was used as a substrate in the TYR activity assay. Additionally, three cataract patients who received Alprazolam (given as preoperative anxiolytic medication) and one patient who was being treated with Sinemet (Carbidopa-Levodopa) for Parkinsonism were excluded from the study.

Melanin biopolymers are synthesized in melanosomes by melanocytes using the melanogenic enzyme TYR. This cuproenzyme catalyzes the initial two steps of melanin synthesis, i.e., converts tyrosine amino acid to DOPA and then to dopaquinone, which is further converted into eumelanin biopolymers. TYR is a glycoprotein mainly localized to melanosome membranes, and a small cohort of the enzyme also exists in soluble form. The characterization of soluble TYR from malignant or normal melanocytes (Wittbjer et al., 1989, Wittbjer et al., 1990a) and bovine eyes (Wittbjer et al., 1990b) has been described. It has been shown that 80% of bovine eye TYR (of size 53 kDa) is soluble and possesses properties similar to those of soluble TYR from human melanoma cells (Wittbjer et al., 1990b). Upon toxicity or damage, iris melanocytes contribute to dispersed pigments in aqueous humor that is possibly enriched with active TYR enzyme in either membrane bound or soluble form. Thus, measuring the activity of TYR in the aqueous is indicative of the extent of melanocyte damage/toxicity. Several methods were developed to measure the activity of soluble TYR present in serum that use radio labeled L-tyrosine, and the investigators measured its conversion to DOPA (Nishioka et al., 1977). In line with these methods, we have developed a non-radioactive based DOPA cytochemistry (Atul Jani et al., 2016) that allows the TYR activity in the aqueous to be measured.

**Fig. 1:**
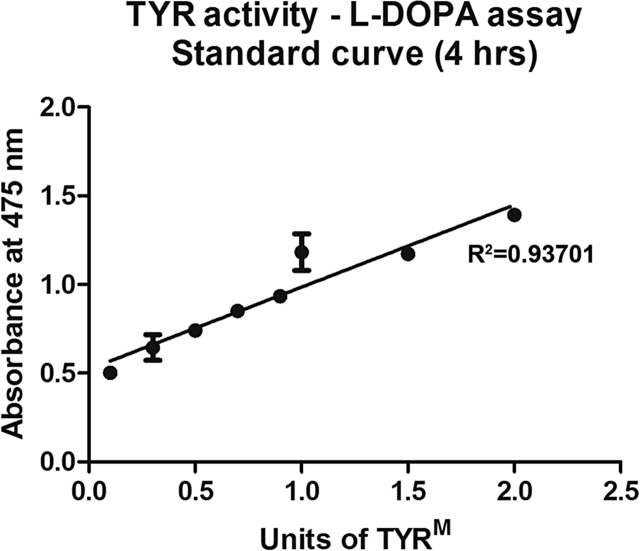
**Plot diagram representing the standard curve of TYR**^**M**^**activity.** Different units of TYR^M^ were incubated with L-DOPA for 4 h, and the melanins were measured as described in Section 2. **R**^**2**^ represents the coefficient of determination. The absorbance value equivalent to 1 U TYR^M^ enzyme was calculated and used to estimate the dispersed pigments in the aqueous humor.

Briefly, the protein concentration of the aqueous (2 μl each in duplicates) was measured using Bradford protein assay reagent (Bio-Rad Laboratories, Hercules, CA, USA) in a 96-well plate. Since untreated (without topical antibiotics) aqueous humor could not be obtained as a control due to ethical restrictions, we chose BSA (bovine serum albumin) as a background control for the TYR activity assay. BSA also served as an indicator of L-DOPA auto-oxidation during the incubation period. A volume equivalent to 5 μg of protein (either in duplicates or triplicates) of aqueous sample or BSA was diluted to 50 μl in phosphate buffer saline (PBS). Further, 100 μl of freshly made 0.1% L-DOPA (Sigma-Aldrich, St. Louis, USA) in PBS was added to the samples, which were incubated at 37 °C for 4 h and had the pigmentation measured at 475 nm using a Tecan Infinite F 200-Multi mode plate reader (Tecan Group Ltd., Switzerland). Sample values (represented as arbitrary units, A.U.) were normalized to their protein concentration (A.U./μg). Finally, the relative aqueous TYR activity (represented as units, U) was calculated by normalizing the absorbance value to the activity of one unit of mushroom TYR (TYR^M^ or TYRM; Sigma-Aldrich, St. Louis, USA) obtained from the standard curve (Fig. 1). An increase in TYR activity in the aqueous above the BSA was considered a possible indicator of dispersed pigments, which is a correlate to the destruction of iris melanocytes.

The standard curve was made using different units of TYR^M^ and L-DOPA as a substrate. TYR^M^ was dissolved in 50 mM potassium phosphate buffer (pH 6.2) at a final concentration equivalent to 1 U/μg protein. After 4 h of incubation, the obtained absorbance values at 475 nm were plotted on the Y-axis (Fig. 1), and the different units of TYR^M^ were plotted on the X-axis. **R**^**2**^, the coefficient of determination, was calculated using Microsoft Excel.

To test whether the observed TYR activity in the aqueous is not due to the auto-oxidation of L-DOPA, a non-specific protein such as BSA (5 μg) was added to the different aqueous samples of Groups A and B, and the TYR activity was measured. Further, to inhibit the aqueous TYR activity, an anti-TYR antibody (human/mouse specific, 6 μg) (Theos et al., 2005) was added to the samples from Groups A and B. As a control, normal rabbit serum was added to the samples before measuring the TYR activity. Note that the anti-TYR antibody used here was not raised against the active site of the TYR protein. It has been shown that FQLs bind melanin and inhibit TYR activity *in vitro* (Beberok et al., 2011, Beberok et al., 2015b). Moreover, studies have shown that concentrations equivalent to 1.13 ± 1.9 μg/ml of Ciprofloxacin (Yalvac et al., 2003) and 1.71 ± 0.82 mg/ml of Moxifloxacin (Halder et al., 2013) were found in the aqueous upon topical application. To study the effects of Moxifloxacin and Ciprofloxacin on TYR activity, a concentration equivalent to 2.5–5000 μg/ml of Vigamox or 1.5–3.0 μg/ml of Ciplox was added to the different samples of Groups A and B separately, and the TYR activity was measured. Finally, to confirm the presence of TYR protein in the aqueous humor, samples equivalent to 20 μg of protein were loaded on SDS-PAGE gel, and immunoblotting was performed as described previously (Jani et al., 2015). As a positive control, mouse melanocyte (Melan-Ink cells) lysate was loaded on the gel and as negative controls, primary human keratinocyte (Invitrogen) or HeLa cell lysate; or BSA or TYR^M^ were loaded on the gel. The immunoblots were probed with polyclonal rabbit anti-TYR antibody sera (Theos et al., 2005). Additionally, an aqueous sample equivalent to 40 μg was concentrated by precipitating with TCA (trichloro acetic acid) using a protocol described earlier (Golemis and Brent, 1997) and then analyzed by immunoblotting.

The average TYR activity of each group was calculated (Table 1). Note that the Moxifloxacin in our group (Group A) was preservative-free and Ciprofloxacin (Group B) and Tobramycin (Group C) had the same preservative concentrations; the comparison between Groups B and C was considered important for commenting on FQL toxicity. The Ciplox^®^ and Tobrex^®^ eye drops used in our study had 0.01% BAK (benzalkonium chloride). However, it has been shown that 0.001% BAK as a preservative does not show any cytotoxicity on ocular cells (Niwano et al., 2014). Statistical significance was determined by an unpaired Student**'**s **t**-test and variance analysis using the GraphPad software (*, *p* < 0.05; **, *p* < 0.01 and ***, *p* < 0.001). All values are described as the mean ± s.e.m.

**Table 1:**
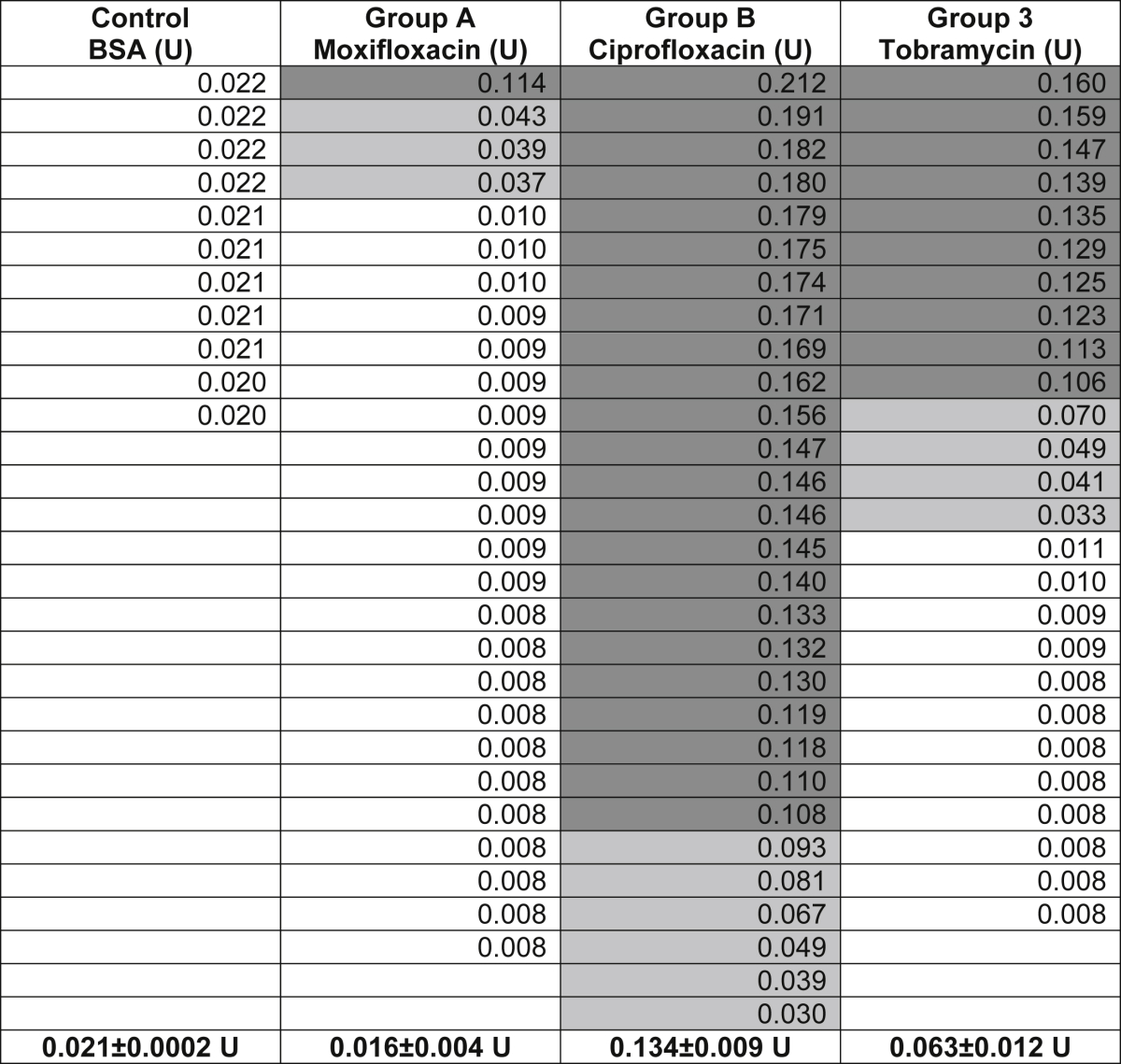
Table containing the individual aqueous TYR activity (at 4 h) in different groups, measured as units (U) of TYR^M^. Each value corresponds to the average TYR activity obtained from duplicate or triplicate samples. Cumulative TYR activity/group is indicated (in bold) separately. The color of the box represents the extent of TYR activity (black for high, gray for low and white for no TYR activity).

## 3 Results

**Fig. 2:**
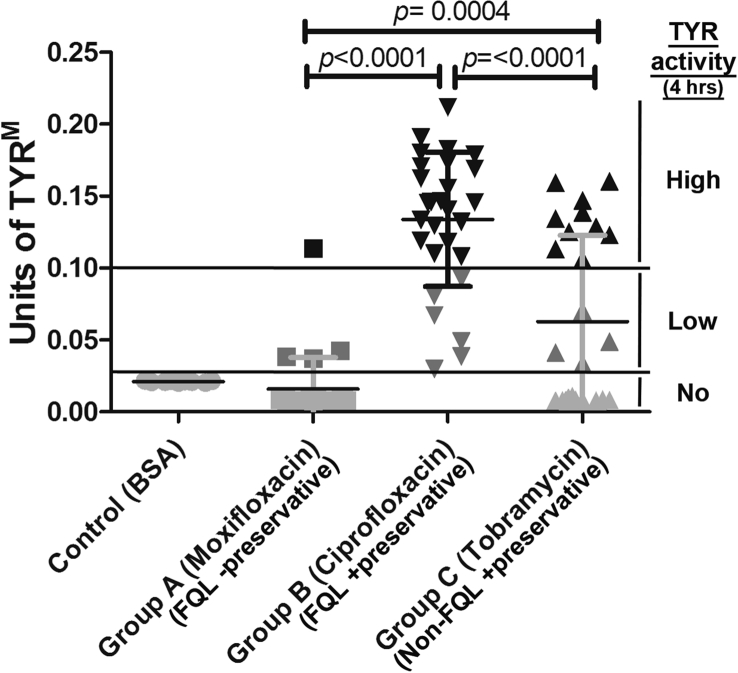
**Plot diagram showing the activity of TYR in aqueous humor samples.** Aqueous TYR activity was measured at 4 h in all patients from Groups A, B and C along with the control BSA. Each data point represents the average activity of duplicate or triplicate samples. Aqueous TYR activity above the BSA level is indicative of the presence of TYR. Likewise, TYR activity below the BSA level represents either the absence of TYR enzyme or its lack of activity in the aqueous. Within the group, a few samples cluster together and show more than 0.1 U of TYR^M^, which specifies the presence of high TYR enzyme in the aqueous, possibly corresponding to high iris melanocyte toxicity. Statistical value *p* < 0.05 = *; *p* < 0.01 = ** and *p* < 0.001 = ***.

In this study, we analyzed TYR activity in the aqueous humor of 82 eyes of 82 cataract patients (Table 1). The average TYR activity (at 4 h) in the aqueous of cataract patients was as follows: Group A (Moxifloxacin): 0.016 ± 0.004 U, Group B (Ciprofloxacin): 0.134 ± 0.009 U and Group C (Tobramycin): 0.063 ± 0.012 U (Table 1 and Fig. 2). In contrast, the control samples (BSA, no TYR) showed activity (reflecting the auto-oxidation of L-DOPA) equivalent to 0.021 ± 0.0002 U at 4 h (Table 1 and Fig. 2). The TYR activity varied from sample to sample within a group (Fig. 2). Aqueous TYR activity above the BSA level is indicative of the presence of TYR. Notably, within the group, few samples showed very high TYR activity, and a cohort displayed low activity (Table 1 and Fig. 2). As observed, few aqueous samples showed TYR activity equivalent to or lower than the control BSA (Table 1 and Fig. 2). Interestingly, all samples in the Ciprofloxacin FQL Group B showed an increase in TYR activity above the control, which suggests that Ciprofloxacin is highly toxic to iris melanocytes (Fig. 2). In contrast, samples in the Moxifloxacin FQL group (except for 4 of the 27) showed no TYR activity (i.e., equivalent to BSA), thus indicating little or no toxicity to iris melanocytes (see below). Unexpectedly, the non-FQL control Tobramycin showed intermediate toxicity to iris melanocytes. Overall, the TYR activity or iris melanocyte toxicity was significantly higher at 4 h in Group B compared to Group C (*p* < 0.0001) or Group A (*p* < 0.0001) (Fig. 2). In the post-operative period, none of the patients developed signs or symptoms of BAIT or BADI. However, we observed certain differences in pigment dispersion in the iris (data not shown), which indicated melanocyte toxicity.

**Fig. 3:**
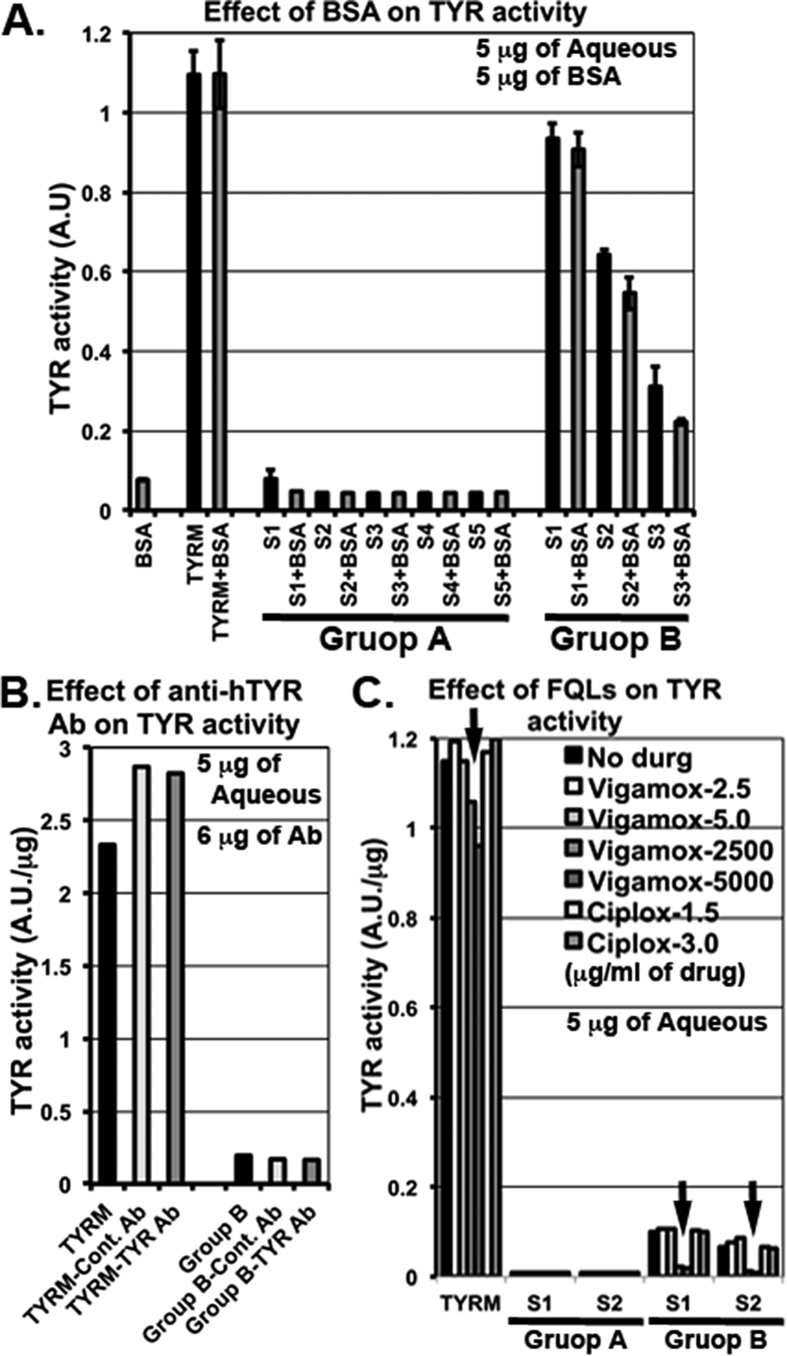
**Plot diagrams showing the effect of non-specific protein, anti-human TYR antibody or FQLs on TYR activity in aqueous humor samples.** Aqueous TYR activity was measured at 4 h in a few samples (S) of Group A (S1 — S5) and Group B (S1 — S3) before and after the addition of 5 μg of BSA (A). TYR^M^ (TYRM) and BSA were used as controls. Note that TYR activity was not affected in Group A samples or TYR^M^, but slightly declined in Group B samples after the addition of 5 μg of BSA, indicating either a sequestration of the aqueous TYR enzyme or a reduction of the conversion of L-DOPA into melanins by BSA. Similarly, TYR activity in Group B samples and TYR^M^ was measured before and after the addition of either control rabbit antibody (Ab) serum or anti-human TYR antibody serum (6 μg of serum) (B). Note that neither control serum nor anti-TYR Ab showed an effect on TYR activity. Likewise, TYR activity in Group A, B samples (S1, S2 each) and TYR^M^ was measured before and after addition of the indicated antibiotics at μg/ml concentration (C). Arrows indicate the reduced TYR activity observed in samples with 2500 or 5000 μg/ml of Vigamox (Moxifloxacin) added including TYR^M^. In all graphs, TYR activity values are presented as arbitrary units (A.U.) or per μg of sample.

**Fig. 4:**
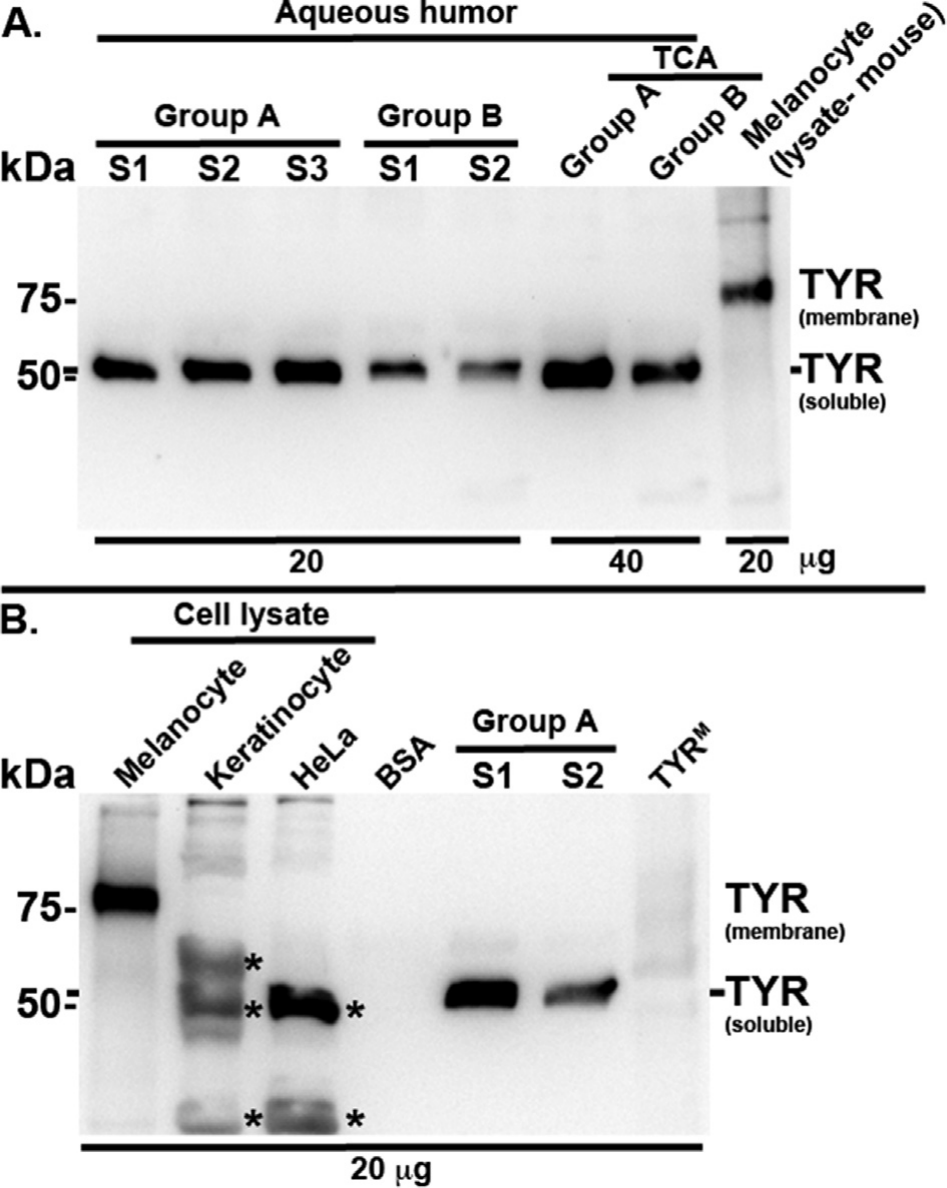
**Immunoblots representing the detection of soluble TYR in aqueous humor.** Samples equivalent to 20 or 40 μg from Group A (S1 — S3), Group B (S1, S2) and TCA precipitated aqueous from Groups A and B were fractionated, immunoblotted and probed with anti-human TYR antibody. Mouse melanocyte lysate was used as a positive control. Note that the anti-TYR antibody detected ∼75 kDa band in mouse melanocyte lysate that corresponds to the melanosome-bound TYR and ∼50 kDa band in all aqueous humor samples, possibly corresponding to soluble TYR, as observed in bovine eye extracts (Wittbjer et al., 1990b). The specificity of the ∼50 kDa band in the aqueous humor was tested by repeating the immunoblotting experiment using cell extracts from human primary keratinocytes and HeLa; BSA or TYR^M^ as negative controls; two samples from Group A and melanocyte lysate (mouse) as positive controls. * indicates the non-specific band detected with the antibody. Note that the anti-TYR antibody could not detect any bands in BSA or TYR^M^, but identified a unique TYR band in the aqueous humor samples.

Interestingly, the TYR activity observed in the aqueous samples was not altered or slightly reduced in samples from Groups A or B respectively, after the addition of non-specific protein such as 5 μg of BSA (possibly inhibiting the activity of aqueous TYR but not TYR^M^) (Fig. 3A) to the aqueous humor. These results indicate that the observed TYR activity is specific to TYR enzyme and may not be due to the auto-oxidation of L-DOPA. Furthermore, the activity of TYR in the aqueous or TYR^M^ was not inhibited with the addition of 6 μg of anti-TYR antibody compared to the presence or absence of control serum (Fig. 3B). This could be due to the absence of binding capacity of the antibody to the active site of TYR enzyme. In contrast, the aqueous TYR activity was significantly inhibited with the addition of 2.5 or 5.0 mg/ml of Moxifloxacin to the aqueous of Ciprofloxacin-treated eyes (Fig. 3C). Similarly, the activity of TYR^M^ was slightly reduced in this condition. However, 2.5–5.0 μg/ml of Moxifloxacin or 1.5–3.0 μg/ml of Ciprofloxacin showed no effect on aqueous TYR or TYR^M^ activity (Fig. 3C). These results clearly indicate that higher concentrations of Moxifloxacin inhibit the aqueous TYR activity. Further, these results explain the lower TYR activity that was observed in the aqueous of Moxifloxacin-treated eye samples. Detection of TYR protein in the aqueous samples by immunoblotting identified a unique band corresponding to a size above 50 kDa in samples from both Groups A and B (Fig. 4A). Consistently, the TCA precipitated aqueous lysates also showed similar results (Fig. 4A). In contrast, the mouse melanocyte lysate that was used as a positive control highlighted a TYR band of the expected size, above 75 kDa (Jani et al., 2015). However, the ∼50 kDa band was completely absent in the negative controls, such as TYR^M^ and BSA (Fig. 4B), indicating that the observed band in the aqueous is specific to TYR. In line with these results, keratinocyte and HeLa cell lysates (human) showed non-specific bands (below or above ∼ 50 kDa) with the anti-TYR antibody (Fig. 4B). These results clearly detected the presence of an altered isoform of TYR (size ∼50 kDa) in the aqueous humor of fluoroquinolone-treated eyes. We predict that this TYR isoform corresponds to the soluble TYR identified in the cell extracts of bovine eyes (Wittbjer et al., 1990b). Nevertheless, the characterization of aqueous TYR requires future investigation using mass spectrometry.

## 4 Discussion

Dermal phototoxicity, cardiotoxicity, arthropathy and tendinitis are known adverse reactions to systemic FQL usage (De Sarro and De Sarro, 2001). Likewise, dispersed pigments, which are the characteristic feature of BAIT and BADI (Tugal-Tutkun et al., 2011), could be a possible phototoxic effect of FQLs on iris melanocytes or iris stroma (Bringas Calvo and Iglesias Cortinas, 2004, Hinkle et al., 2012, Wefers Bettink-Remeijer et al., 2009, Willermain et al., 2010). However, BAIT but not BADI has been described as an ocular side effect of systemic FQLs (Hinkle et al., 2012). In addition, emerging viral etiology has been suspected for both BADI and BAIT (Tugal-Tutkun et al., 2009, Tugal-Tutkun et al., 2011, Tugal-Tutkun and Urgancioglu, 2006). Nevertheless, the accumulation of dispersed pigments in the aqueous or the occurrence of BAIT or BADI upon the topical use of FQLs has never been tested.

The effects of FQLs on dermal melanocytes have been studied in detail. FQLs such as Norfloxacin, Moxifloxacin (Beberok et al., 2015b), Sparfloxacin (Beberok et al., 2015a) and Ciprofloxacin (Beberok et al., 2011) are known to affect both human skin melanocyte viability and TYR activity *in vitro*. Studies have also reported an increase in aqueous TYR activity in melanoblastoma (Kloucek and Matous, 1972). However, studies on the effect of FQL on iris depigmentation and melanocyte toxicity *in vivo* have not been reported. We hypothesize that FQL may cause iris melanocyte toxicity and result in the release of dispersed pigments into aqueous humor and that these products can be detected in slit-lamp biomicroscopy but not quantitatively. Melanins are synthesized by membrane bound melanogenic TYR enzyme within the melanosomes (Raposo and Marks, 2007, Sitaram and Marks, 2012). We predict that the dispersed pigments present in the aqueous are also enriched with TYR enzyme and that their activity can be measured using L-DOPA assay (Atul Jani et al., 2016). Thus, for the first time, we have standardized (Fig. 1) and quantified the TYR activity in the aqueous of 82 healthy eyes undergoing cataract surgery (Fig. 2 and Table 1). Our studies show that the topical medication of FQLs has different effects on aqueous TYR activity (Fig. 1). The increase in aqueous TYR activity (upon incubation with L-DOPA *in vitro*) possibly indicates the extent of iris melanocyte destruction by FQLs and the resulting release of TYR or dead melanocytes into the aqueous, which corresponds to the dispersed pigments/melanocyte debris. We have measured the activity of aqueous retained TYR using L-DOPA as substrate and incubated the reaction for 2–4 h. We found that TYR activity in the aqueous consistently increased from 2 h and became saturated at 4 h (data not shown). Further, our study showed that TYR activity (at 4 h) is significantly higher in the aqueous of eyes treated with Ciprofloxacin FQL (Group B) compared to non-FQL Tobramycin (Group C; *p* < 0.0001) or another FQL Moxifloxacin (Group A; *p* < 0.0001). Moreover, our results with Ciprofloxacin are consistent with an earlier report on dermal melanocytes (Beberok et al., 2011). Note that the Moxifloxacin (Vigamox^®^) used in our study does not contain any preservatives, unlike Tobramycin or Ciprofloxacin, which contain a similar concentration of BAK as preservative. However, the effect of 0.001% BAK on ocular cells shows no toxicity (Niwano et al., 2014) and the drugs used here contain 0.01% BAK, which may not cause iris melanocyte toxicity compared to the antibiotic concentrations used during phacoemulsification. Furthermore, the observed TYR activity in the aqueous is not due to the auto-oxidation of the L-DOPA substrate but is specific to the presence of TYR protein (Fig. 4) in the sample. Thus, the TYR activity results of Moxifloxacin-treated eye aqueous samples surprised us in two ways: (1) the TYR activity values were always very low compared to another member of the same family, Ciprofloxacin, and (2) the presence of TYR enzyme was detected in the samples. We hypothesized that the presence of Moxifloxacin (higher concentration) possibly inhibits the aqueous TYR activity and also toxic to iris melanocytes. Our further investigations showed a mg/ml concentration of Moxifloxacin (Vigamox) but not a μg/ml concentration of Vigamox or Ciplox inhibited TYR activity (Fig. 3). These results suggest that Moxifloxacin is toxic to the iris melanocytes and releases the dispersed pigments enriched with TYR into the aqueous but that its activity is inhibited possibly by the presence of Moxifloxacin. Our hypothesis is consistent with the presence of 1.71 ± 0.82 mg/ml of Moxifloxacin in the aqueous upon topical application (Halder et al., 2013). In contrast, 1.13 ± 1.9 μg/ml of Ciprofloxacin was estimated in the aqueous (Yalvac et al., 2003), which did not show any TYR activity inhibition in our assay. Our prediction is also consistent with a slightly higher amount of TYR enzyme (indicating more iris melanocyte toxicity) present in the aqueous of Moxifloxacin-compared to Ciprofloxacin-treated samples (Fig. 4). Importantly, the non-FQL family member Tobramycin also caused melanocyte toxicity/dispersed pigments in a few aqueous samples compared to the preservative-free Moxifloxacin or the BSA control. Tobramycin is a member of the aminoglycoside family, which has also been shown to have strong binding with melanin and has been speculated to be involved in ototoxicity (Buszman et al., 2007, Wrzesniok et al., 2013).

Interestingly, the aqueous TYR activity within the group is quite variable possibly due to a disparity in temporal effect or variability in the effective concentration of antibiotics on iris melanocyte toxicity among the patients, in addition to the amount of antibiotic accumulated in aqueous. Alternate technical possibilities would include effects due to batch variation in sample storage or transportation from clinic to the lab. Moreover, our study did not account for the effects of medications that are used for diabetes, hypertension and cardiac ailments on aqueous TYR activity, which are unknown to us. Additionally, we were unable to use a healthy patient's untreated (without antibiotics) aqueous sample as a control for the drug treatment due to restrictions on our ethical practice. However, our study for the first time confirmed the presence of TYR in the aqueous humor, which contributes to the activity by converting L-DOPA to melanin. Interestingly, the aqueous TYR band detected in our immunoblots did not show the expected size of human/mouse membrane-bound TYR (∼75 kDa). In contrast to skin melanocytes, several studies have reported the presence of soluble TYR in malignant melanoma or bovine eye extracts and detected a band size equivalent to 53 kDa (Wittbjer et al., 1989, Wittbjer et al., 1990a, Wittbjer et al., 1990b). Based on these studies, we hypothesized that the detected band (above 50 kDa) in our western blot corresponds to the soluble TYR. This proposal is consistent with our observed results: (1) a single TYR band was detected in aqueous of both Moxifloxacin- and Ciprofloxacin-treated eye samples, and (2) no band other than this unique band was observed, even after the concentration of aqueous samples (Fig. 4). Additionally, bovine eye extract has been shown to contain 80% of soluble TYR (Wittbjer et al., 1990b). Based on the results, we predict that iris melanocytes possibly express higher amounts of soluble TYR compared to membrane-bound TYR, which we did not detect in our experiments. Moreover, characterizing the soluble TYR in the aqueous by mass spectrometry is essential to confirm the detected TYR band in our western blots. Nevertheless, the expression levels, trafficking, localization and function of the soluble TYR in iris melanocytes would be of interest to study in the future.

In this study, we have also tested whether the topical application of FQL will cause any clinically appreciable iris changes. Interestingly, none of our patients showed any ocular side effects suggestive of BAIT or BADI phenotypes. Thus, our results suggest that direct FQL exposure causes subclinical toxicity to iris melanocytes, which results in pigment release into the aqueous but may not be the sole cause of acquired atrophic iris diseases (BAIT/BADI). This method detected TYR and its activity in the aqueous humor *in vivo*, and it shows promise as a potential diagnostic test to measure the toxicity of drugs applied during eye diseases.

### 4.1 Conclusions

Iris melanocytes generally release melanins or dispersed pigments into the aqueous humor due to ocular side effects during pathogenesis or the topical application of antibiotics. Although procedures are available to detect these pigment deposits/melanocyte debris in the aqueous, no method was previously available to quantify them. Here, we have developed an L-DOPA-based TYR activity assay to measure the dispersed pigments in the aqueous humor, which corroborate the extent of melanocyte damage or toxicity during treatment. This method can be utilized to quantify iris damage due to various diseases or drug toxicity. Moreover, our study, for the first time, showed the presence of active and soluble TYR in the aqueous of FQL-treated patients. In conclusion, the mere topical application of FQL does not result in development of BAIT or BADI in cataract patients in pre- or post-operative periods, but it can cause subclinical iris melanocyte toxicity *in vivo*.

## Financial disclosures

No financial disclosures.

## Competing interests

The authors have no competing interests.

## Conflicts of interest

Nil.

## Author contributions

S.M., A.A.K., P.M. and S.R.G.S. design the strategy and experimentation. S.M. and S.S.D. standardized and performed the tyrosinase activity assays. M.K. and V.K. contributed by collecting aqueous sample from anterior chamber. A.A.K. and P.M. performed the clinical studies. All authors contributed to the data acquisition. S.M., A.A.K. and S.R.G.S. analyzed the data including statistics. A.A.K., S.M. and S.R.G.S. wrote the manuscript. S.R.G.S. coordinated and discussed the work with co-authors.
